# Novel Victorivirus from a Pakistani Isolate of *Alternaria alternata* Lacking a Typical Translational Stop/Restart Sequence Signature

**DOI:** 10.3390/v11060577

**Published:** 2019-06-25

**Authors:** Atif Jamal, Yukiyo Sato, Sabitree Shahi, Wajeeha Shamsi, Hideki Kondo, Nobuhiro Suzuki

**Affiliations:** 1 Institute of Plant Science and Resources, Okayama University, Chuou 2-20-1, Kurashiki, Okayama 710-0046, Japan; y-sato@okayama-u.ac.jp (Y.S.); ss.savitri.shahi@gmail.com (S.S.); wajeeha.shamsi@gmail.com (W.S.); hkondo@okayama-u.ac.jp (H.K.); 2 Crop Diseases Research Institute, National Agricultural Research Centre, Park Road, Islamabad 45500, Pakistan

**Keywords:** mycovirus, victorivirus, *Totiviridae*, dsRNA, stop/restart translation, pseudoknot structure, *Alternaria alternata*, *Cryphonectria parasitica*, Indian subcontinent

## Abstract

The family *Totiviridae* currently contains five genera *Totivirus*, *Victorivirus*, *Leishmavirus*, *Trichomonasvirus*, and *Giardiavirus*. Members in this family generally have a set of two-open reading frame (ORF) elements in their genome with the 5′-proximal ORF (ORF1) encoding a capsid protein (CP) and the 3′-proximal one (ORF2) for RNA-dependent RNA polymerase (RdRp). How the downstream open reading frames (ORFs) are expressed is genus-specific. All victoriviruses characterized thus far appear to use the stop/restart translation mechanism, allowing for the expression of two separate protein products from bicitronic genome-sized viral mRNA, while the totiviruses use a −1 ribosomal frame-shifting that leads to a fusion product of CP and RdRp. We report the biological and molecular characterization of a novel victorivirus termed Alternaria alternata victorivirus 1 (AalVV1) isolated from *Alternaria alternata* in Pakistan. The phylogenetic and molecular analyses showed AalVV1 to be distinct from previously reported victoriviruses. AalVV1 appears to have a sequence signature required for the −1 frame-shifting at the ORF1/2 junction region, rather than a stop/restart key mediator. By contrast, SDS–polyacrylamide gel electrophoresis and peptide mass fingerprinting analyses of purified virion preparations suggested the expression of two protein products, not a CP-RdRp fusion product. How these proteins are expressed is discussed in this study. Possible effects of infection by this virus were tested in two fungal species: *A. alternata* and RNA silencing proficient and deficient strains of *Cryphonectria parasitica*, a model filamentous fungus. AalVV1 showed symptomless infection in all of these fungal strains, even in the RNA silencing deficient *C. parasitica* strain.

## 1. Introduction

There are many double-stranded RNA (dsRNA) viruses with a two-open reading frame (ORF), nonsegmented genome organization, one of the simplest genome types, reported from plants, fungi, protozoa, and insects, as exemplified by totiviruses (members of the genus *Totivirus*) and members of other genera in the *Totiviridae* [1], amalgaviruses [2,3], phlegiviruses [4], fusagraviruses [5], megatotiviruses [6], and many unclassified viruses. The genome sizes of such viruses range from 3.5 (amalgaviruses) to ~12 kbp (megatotiviruses). With some exceptions such as amalgaviruses and phlegiviruses, these viruses typically encode capsid protein (CP) on the 5′-proximal ORF and RNA-dependent RNA polymerase (RdRp) on the 3′-proximal ORF. The 3′ proximal ORFs of these viruses are expressed by noncanonical translation mechanisms such as stop/restart translation [7,8] and ribosomal frame-shifting [9,10,11], though not substantiated, for many of these viruses. These viruses are expected have icosahedral *T* = 1 capsids as shown for totiviruses [12,13].

Members of the family *Totiviridae*, comprising five genera (*Totivirus*, *Victorivirus*, *Leishmaniavirus*, *Trichomonasvirus*, and *Giardiavirus*), are characterized by undivided dsRNA genomes with two ORFs encoding a CP and an RdRp or RdRp domain of a fusion protein. Among them, most members of which (belonging to genera *Totivirus* and *Victorivirus*) are isolated from fungi. Most totiviruses (members of the genus *Totivirus*) characterized thus far have been reported from yeast as exemplified by Saccharomyces cerevisiae virus L-A (ScV-L-A). However, recently totiviruses were reported from obligate filamentous phytopathogenic fungi [14] and plants [15] (see [16] for review). The characterized victoriviruses were from filamentous fungi but not from yeast. Members of the two genera are different from each other in several features such as the translation strategy and phylogenetic placement. Yeast totiviruses snatch the 5′ cap structure from host mRNAs and the snatching activity resides in the CP and is then mapped to His-154 that is located in the trench on the outer-surface or the cytoplasmic surface of the virion [17,18]. Note that SsV-L-A is the first dsRNA fungal virus whose 3-D structure model of *T* = 1 capsids with 120 homodimers was proposed [12,19,20]. The 5′ proximal ORF is translated according to the scanning model while the 3′-proximal ORF is expressed as a CP-RdRp fusion product by the −1 ribosomal frame-shifting mediated by slippery sequences and downstream pseudoknot or stem-loop structures. The rate of the frame-shifting ribosomes was estimated as 1.9% of ribosomes that have translated CP [11].

Like totiviruses, victoriviruses form icosahedral particles of ~40 nm in diameter with a *T* = 1 lattice composed of 60 CP dimers (120 molecules) [21]. Victoriviruses appear to have internal ribosomal entry sites (IRESs) in the 5′ untranslated region for the CP translation [22], while the RdRp is translated from bicistronic viral mRNA by the stop/reinitiation or coupled termination/reinitiation mechanism as a separate product from CP, not a fusion product, unlike totiviruses [7,8]. The reinitiation in victoriviruses is most likely to be mediated largely by the “*AUG*A” or “UA*A**UG*” [7,8], in which the underlined triplet serves as the stop codon for the CP ORF while the italicized triplet is the initiation codon for the RdRp ORF. The UA*A**UG* is also used by the well-studied prototypic hypovirus Cryphonectria hypovirus 1 (CHV1, a single-strand RNA virus) [23]. Li et al. identified two RNA sequence elements, the *AUG*A motif combined with an upstream pseudoknot RNA structure, as the necessary sequence elements for the stop/restart translation of victoriviruses [8]. Therefore, members of the two genera are different in the translation strategy for both the first and second ORFs.

Almost all victorivirus show symptomless infections. Exceptions include Helminthosporium victoriae virus 190S (HvV190S) that induces debilitation in the natural fungal host (*Helminthosporium victoriae*) and an RNA silencing mutant of *Cryphonectria parasitica* [24], a model filamentous fungus for mycovirus research [25]. Another victorivirus from *Rosellinia necatrix* (Rosellinia necatrix victorivirus 1, RnVV1) causes no overt phenotypic alterations in the natural host and the standard strain of *C. parasitica* [26]. However, RnVV1 induces a growth defect in an antiviral RNA silencing deficient strain in which the antiviral host defense is compromised [26]. Interestingly, RnVV1 is very susceptible to RNA silencing and eliminated by coinfection with another RNA mycoviruses or transgenic expression of the hairpin dsRNA, both of which can induce host antiviral RNA silencing [27].

Here we report the molecular and biological characterization of a victorivirus strain, which was isolated from a field strain, A-16, of *Alternaria alternata*, a phytopathogenic ascomycete, isolated from a wheat grain in Pakistan. The virus was tentatively termed Alternaria alternata victorivirus 1 (AalVV1). Surprisingly, the virus lacks a typical stop/restart signature sequence for the translation of RdRp, thought to be the rule for victoriviruses, but instead likely has a sequence feature often found for the −1 frame-shifting at the ORF1 and ORF2 junction. This paper represents the second report on a fungal virus from the Indian subcontinent.

## 2. Materials and Methods

### 2.1. Fungal Culture and Isolation Condition

The fungal strain designated as A-16 was isolated from a wheat grain at the National Agricultural Research Centre (NARC), Islamabad, Pakistan by a standard blotter method as described by the International Seed Testing Association) ISTA [28].

The strain was identified as *A. alternata* based on morphological characteristics of spores and on the sequence of the internal transcribed spacer (ITS) regions of the fungal ribosomal DNA (rDNA) amplified by using a primer pair ITS1 (TCCGTAGGTGAACCTGCGG) and ITS4 (TCCTCCGCTTATTGATATGC) [29]. The culture was maintained on potato dextrose agar (PDA, BD Difco Laboratories, Detroit, MI, USA) at 25 °C and for RNA extractions (small and large scale), mycelial plugs were inoculated in potato dextrose broth (PDB, BD Difco Laboratories) at 25 °C for two weeks. To extract dsRNA, mycelial plugs were grown in PDB under shaking (150 rpm) at 25 °C for seven days for mycelial mass collection [30]. A virus-free Japanese strain, Ally-12 of *A. alternata* f. sp. *lycopersici* [31], was provided by Dr. Masatoki Taga, Okayama University, while the standard strain EP155 of *C. parasitica* and its mutant ∆*dcl2* [32] were a generous gift from Dr. Donald L. Nuss, University of Maryland.

### 2.2. RNA Preparation

Total nucleic acid was extracted from the mycelia of the fungal strain A-16 cultured in 20 mL PDB while the dsRNA fraction was isolated from mycelia grown on PDA overlaid with cellophane (PDA-cellophane) until it covered the plate [30]. Harvested mycelia were ground in mortar with liquid nitrogen followed by the addition of buffer (100 mM Tris-HCl pH 8.0, 4 mM EDTA, 200 mM NaCl and 2% SDS) and clarification with one round each of phenol-chloroform and chloroform in 2ml tubes. Total nucleic acid fractions were obtained by ethanol precipitation. For dsRNA isolation, the resulting extract was mixed with 16% ethanol, STE buffer (10 mM Tris-HCl pH 8.0, 1 mM EDTA and 150 mM NaCl), and 0.05 g of cellulose powder with 200–300 mesh (Advantech, Tokyo, Japan), followed by one hour of incubation with continuous rotation at room temperature. The sample was washed thrice with STE and ethanol (16%), vortexed and centrifuged between washes and the resulting dsRNA was eluted once with STE buffer from dried cellulose powder and precipitated with ethanol and sodium acetate. The sample was analyzed using 1% agarose gel electrophoresis. A fragment of approximately 5 kbp was observed and the dsRNA nature was confirmed after treatment with RNase free DNase I and S1 nuclease (Takara, Shiga, Japan).

### 2.3. Viral Genome Sequencing using RNA-Seq

An equal amount of dsRNA samples prepared from three virus infected strains of different fungi including *A. alternata* strain A-16 and unidentified fungi were pooled (namely “pool A1”) and 5.9 µg of dsRNA (91 ng/µL) was subjected to next generation sequencing. Viruses detected from the other two strains of the fungi will be reported elsewhere. The cDNA library construction and subsequent deep sequencing on the Illumina platform (HiSeq 2500, 50 bp single-end reads) were carried out by Macrogen Inc. (Tokyo, Japan). After sequencing, the adaptor sequences are trimmed and then the adaptor-trimmed reads (29,875,302 reads) were assembled *de novo* into 14,209 contigs (~488–6785 nt in length) using CLC Genomics Workbench (version 11, CLC Bio-Qiagen, Aarhus, Demark). These contigs were subjected to local BLAST searches against the viral reference sequence (RefSeq) dataset of the National Center for Biotechnology Information (NCBI). The complete nucleotide sequence of the virus genome in this article has been deposited with the EMBL/GenBank/DDBJ Data Library under Accession No. LC477336.

### 2.4. Database Search and Sequence Analysis

Potential ORFs were identified using the EnzymeX version 3.3.3 [33] Sequence similarities were calculated using the protein BLAST program (blastp) available from NCBI. The RNA secondary structure (pseudoknots) was predicted using the DotKnot program [34] and drawn with PseudoViewer3 [35].

### 2.5. Phylogenetic Analysis

Phylogenetic analysis was based on a maximum likelihood (ML) method as described previously [36] with minor modifications. Virus sequences were aligned with the MAFFT version 7 [37] and subsequently ambiguously aligned regions were eliminated using Gblocks 0.91b [38]. We then used PhyML 3.0 [39] to construct the ML phylogenetic trees with the automatic model selection by Smart Model Selection (SMS) [40]. The phylogenetic tree was visualized and refined with the FigTree graphical viewer interface version 1.3.1 [41].

### 2.6. RT-PCR for Confirmation of Contigs and RLM-RACE for Terminal Sequence Determination

Reverse transcription (RT)-polymerase chain reaction (PCR) was carried out to confirm that contig (A1-19) was derived from the genome of a victorivirus. Approximately 10 µg of purified dsRNA along with 10 pmol random hexamer (N6) was denatured by dimethyl sulfoxide (DMSO) and reverse transcribed at 37 °C for an hour in a 20-µL reaction mixture containing 50 mM Tris-HCl (pH 8.3), 50 mM KCl, 4 mM MgCl_2_, 10 mM dithiothreitol, 40 U of moloney murine leukemia virus reverse transcriptase (Thermo Fisher Scientific, Invitrogen, Waltham, MA, USA), and 20 U of RNase inhibitor (Toyobo, Osaka, Japan). One µL of the reaction mixture was subjected to PCR with sequence-specific primer sets, AJ-19F (1)/AJ-19R (1) and AJ-19F (2) and AJ-19R (2), respectively (Table S1).

For determination of the terminal sequences 5′ and 3′, 3′ RNA ligase-mediated amplification of cDNA ends (RLM-RACE) was done according to the procedure described by Suzuki et al. [42] with slight modifications. A 5′-phosphorylated, 3′-amino-linked oligodeoxy-nucleotide (LIG-Rev, 5′-PO_4_-GATCCAACTAGTTCTAGAGCGG-NH_2_-3′) was ligated to the 3′-end of the dsRNA using T4 RNA ligase (Takara) at 16 °C for 16 h. The ligated dsRNA was then denatured and used as the template in the first strand cDNA synthesis at 42 °C for one h in which an oligo-deoxynucleotide (LIG-FOR, 5′-CCGCTCTAGAACTAGTTGGATC-3′), complementary to the adapter, and Superscript II RNase H-Reverse Transcriptase (Thermo Fisher Scientific) were used. Resulting cDNA was used for PCR with sequence-specific primer (Table S1) along with primer LIG-FOR. PCR products were cloned and then sequenced using the Sanger sequencing method.

### 2.7. Purification of Virus Particles

Viral particles were purified as described by Shamsi et al. [43]. This preparation was used for molecular analysis, transfection, and electron microscopy. For electron microscopy (EM) observations, purified virus particles were negatively strained with an EM stain (EM stainer, an alternative for uranyl acetate, Nissin EM Co., Tokyo, Japan). Prepared specimens were observed in a Hitachi model H-7650 transmission electron microscope (Hitachi, Tokyo, Japan) and photographed as digital images. SDS–polyacrylamide gel electrophoresis (PAGE) analysis followed with Coomassie Brilliant Blue (CBB) and silver staining was also performed as previously described [43].

The protein bands were silver-stained and subjected to in-gel tryptic digestion followed by liquid chromatography–tandem mass spectrometry (LC-MS/MS) analysis as described by Shamsi et al. [43].

### 2.8. Transfection of C. parasitica Spheroplasts

Transfection of *C. parasitica* was performed as described previously [44,45]. Spheroplasts of the *C. parasitica* Δ*dcl2* strain were prepared using the method of Eusebio-Cope et al. [30]. A-16 (a victorivirus) virions were purified according to the method described above by differential and cesium chloride (CsCl) gradient centrifugation. Purified particles were transfected into *C. parasitica* Δ*dcl2* spheroplasts using polyethylene glycol (PEG). After regeneration of the spheroplasts, mycelial plugs from multiple positions were transferred onto new PDA plates and propagated. Transfectants were analyzed by agarose gel electrophoretic analysis of their dsRNA-enriched fractions.

### 2.9. Protoplast Fusion

Protoplasts of the Japanese *A. alternata* strain Ally-12 were prepared with the method as described by Shahi et al. [46] and transformed by pCPXHY3, carrying a hygromycin resistance gene (hygromycin B phosphotransferase), to obtain a hygromycin-resistant (HygR) strain. The resulting transformant was used as a recipient, while the Pakistani strain A-16 (carrying a victorivirus) was used as a donor. Equal amounts of protoplast (2 × 10^7^) from strains Ally-12 and A-16 were mixed and fused in the presence of PEG. Fungal colonies showing a HygR phenotype were tested for virus infection.

## 3. Results

### 3.1. Genome Organization of a Victorivirus Alternaria alternata victorivirus 1

**Figure 1:**
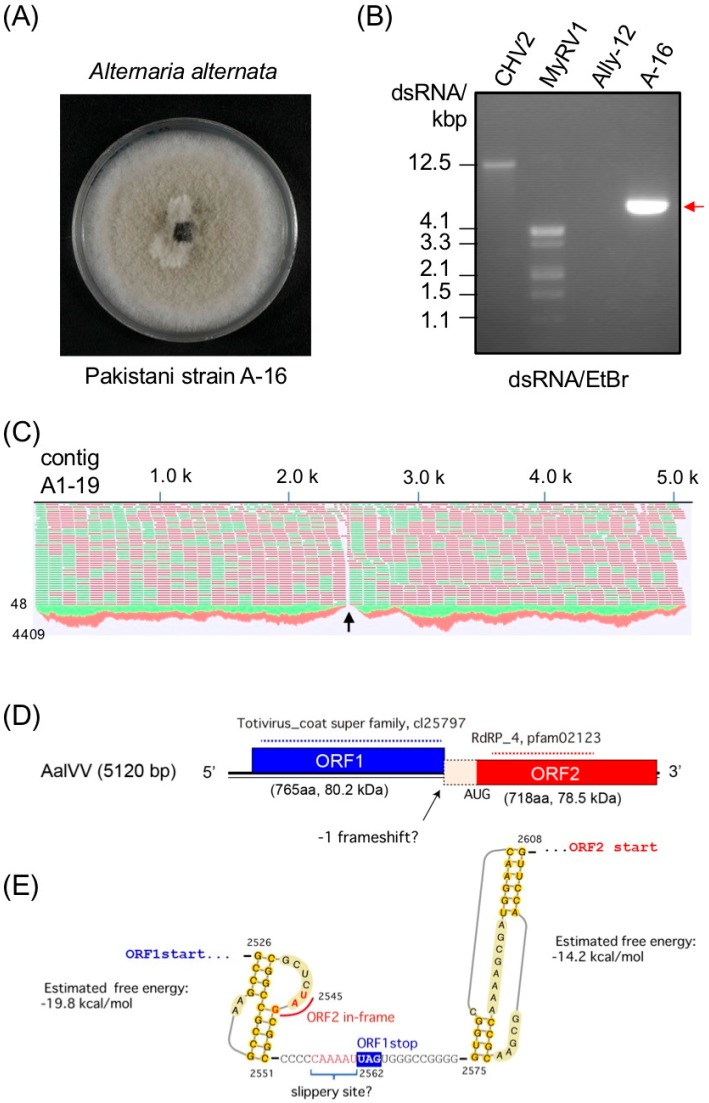
Molecular characterization of Alternaria alternata victorivirus 1 (AalVV1). (**A**) Colony morphology of a Pakistani *Alternaria alternata* strain A-16. The fungal isolate was grown on potato dextrose agar for one week and photographed. (**B**) Agarose gel electrophoresis of AalVV1 (A-16) genomic dsRNA. AalVV1 was separated on 1.0% agarose gel and stained with ethidium bromide (EtBr). The AalVV1 genomic dsRNA of 5.1 kbp is shown by a red arrow. (**C**) Read mapping of the contig A1-19 (AalVV1). The reads are differentiated by strands with red and green, respectively. A region with significantly low read coverage is shown by a black arrow (**D**) Genomic organization of AalVV1. The two nonoverlapping open reading frames (ORFs) and the untranslated regions (UTRs) are shown by red and blue boxes and a black line, respectively. ORF 1 (765aa) encodes capsid protein (CP) of 80.2 kDa, whereas ORF2 (718aa) encodes an RNA dependent RNA polymerase (RdRp). The putative slippery site for the −1 frameshift (boxed) is shown at the junction of the two ORF’s with a black arrow along with the start codon of the second ORF. (**E**) Schematic representation of the translation strategy of the downstream ORF (ORF2) employed by AalVV1. Predicted −1 frameshift site, a putative slippery region (blue bracket) at the junction of the two ORF’s at map positions 2556 to 2560 with two possible H-type pseudoknots structures (2526–2551 and 2575–2608) up- and downstream of the termination codon, respectively, along with the estimated free energy for both structures.

A Pakistani fungal strain A-16 (Figure 1A) which carries a mycovirus-like dsRNA element (Figure 1B) was identified as *Alternaria alternata* morphologically and molecularly. The nucleotide sequences of the internal transcribed spacer (ITS) regions of A-16 were compared using [47], and were found to be matched best with the already deposited sequences of *A. alternata* (KM233267, identity: 94.6%).

The raw next generation sequencing (NGS) reads obtained by the HiSeq sequencing were assembled into different contigs. A local BLAST analysis showed the presence of a virus-like contig (A1-19), with an average coverage of 2680 (total 138,061 reads), and a size of 5159 bp in length (Figure 1C), which corresponded well to the length of the dsRNA purified from A-16. A NCBI BLASTn search of the 5159-bp contig revealed significant similarity to the complete genome sequence of Nigrospora oryzae victorivirus 1 (NoVV1, accession number KT428155) (*E*-value, 0; nt identity 68.3%) and Phomopsis vexans RNA virus (PvRV, accession number KP090346) (E-value, 0; nt identity 68.3%), which are unclassified dsRNA mycoviruses that belong to the genus *Victorivirus*. The nucleotide sequence of this putative mycovirus, which we called “Alternaria alternata victorivirus 1” (AalVV1) has been deposited in the GenBank/EMBL/DDBJ databases under accession number LC477336.

RNA ligase-mediated amplification of cDNA ends (RLM-RACE) and RT-PCR analyses revealed that the AalVV1 genomic dsRNA was 5120 bp long and contained two overlapping open reading frames (ORFs) on its plus-strand (Figure 1D). ORF1 was 2298 nt long (positions 267 to 2564 nt) and was predicted to encode a protein with a calculated molecular mass of 80.2 kDa. ORF2 was 2157 nt long (positions 2887 to 5043 nt) and could encode a 78.5-kDa protein (Figure 1D). The 5′- and 3′- untranslated regions (UTR) were found to be 266 nt and 77 nt long, respectively. A tandem stem loops was predicted upstream of the AalVV1 ORF2 (Figure 1E). Although there is no clear evidence for conserved sequences at the genome termini of different victoriviruses [48], the 5′ UTR of AalVV1 showed conservation in a few nucleotides at the beginning of the 5′ UTR with NoVV1 and PvRV respectively, whereas in the 3′-UTR, a small stretch of the conservation with NoVV1 was also observed. In the 5′-UTR, the octanucleotide sequence 5′-AGGGUUCC-3′, which is conserved in several victoriviruses, including Coniothyrium minitans RNA virus (CmRV, accession number AF527633) and Epichloe festucae virus 1 (EfV1, accession number AM261427), was found at nt 204–211 in the AlaVV1, 55 nt upstream of the AUG initiation codon of ORF1. Interestingly, instead of the octanucleotide, an extra seven conserved nucleotides (5′-AGGGUUCC**GUUGAUC**-3′, bold letters) were noted in AalVV1 and several other victoriviruses (Figure S1). One of the victoriviruses (CmRV) has this fifteen nucleotide sequence at the start of the 5′-UTR. These sequences might have an important role to play as they are found in the majority of the victoriviruses (Figure S1A, boxed). The victorivirus CPs are unique in the family *Totiviridae* in having an Ala/Gly/Pro-rich region predicted near their C-termini [48]. The C-terminal sequence of AalVV1 showed the similar Ala/Gly/Pro-rich region as shown in the prototype victorivirus Helminthosporium victoriae virus 190S (HvV190S) (Figure S1C).

Victoriviruses generally utilize stop/restart strategies to translate the RdRp-encoding ORF2 whose in which *AUG*A or UA*A**UG* facilitate it together with pseudoknot structures located at immediately upstream of the facilitators [7,8]. Visual examination failed to detect cotranslational reinitiation facilitators in AalVV1, while a H-type pseudoknot structure was predicted upstream of the CP stop codon by the DotKnot program (Figure 1E). Instead, a possible -1 frameshift slippery site might present at the positions 2545 to 2562 followed by a second pseudoknot structure (Figure 1E). However, no typical heptanucleotide slippery sequence “X XXY YYZ” (spaced triplets represent preframeshift codons), which is a key signal element for -1 ribosomal frame-shifting [49], was found in the region (Figure 1E). However, a slippery-like sequence, “C CaA AAU,” was detected at the nt positions 2555–2561. RT-PCR fragments spanning the ORF1 and ORF2 junction, where the NGS read coverage was significantly low (Figure 1C, arrow), were cloned and sequenced. All six of the RT-PCR clones sequenced conformed to the genetic organization discussed above.

**Figure 2:**
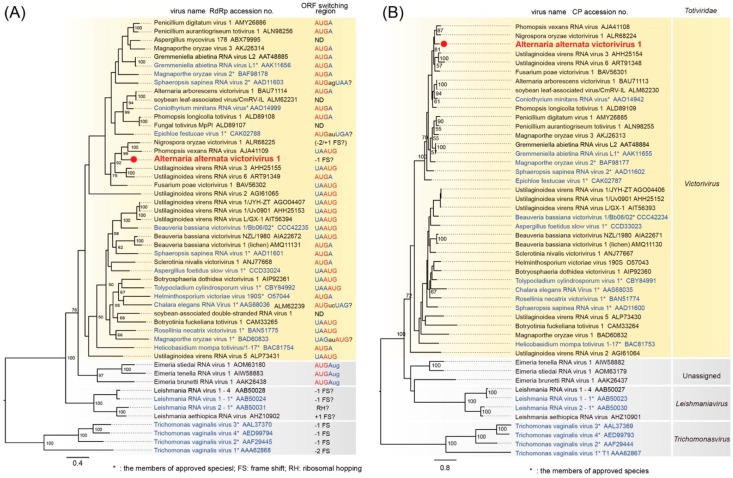
Maximum likelihood (ML) phylogenetic trees based on the RdRp (**A**) and CP sequences (**B**) from members of the genus *Totivirus* and related viruses. The unrooted ML trees were generated by using PhyML 3.0 [39]. The virus names (blue, approved virus species) and their GenBank accession numbers, and the potential translational stop/restart sequences or the ORF switching modes (frame-shifting) are shown in the trees. The selected members of the genera *Leishmaniavirus* and *Trichomonasvirus*, infecting protozoan parasites, were used as the outgroups. The numbers on the branches are the bootstrap values (only values greater than 50% are shown).

BLASTp searches of the deduced amino acid (aa) sequences of ORF1 of the contig A1-19 dsRNA revealed the highest identity, 69.8% (*E*-value, 0.0), to the putative CP of PvRV (Table 1). ORF2 had the highest identity, 61.4% (*E*-value, 0.0), to the putative RdRp of NoVV1 (Table 1). The aa sequence analysis of the putative RdRp of the AalVV1 revealed the presence of eight motifs that were conserved in the RdRps of the members of the family *Totiviridae* (Figure S1B). Accordingly, phylogenetic analysis based on the aligned motif sequences in RdRp showed that AalVV1 clustered together with victoriviruses (genus *Victorivirus*) and was separated from the members of the genera *Leishmaniavirus*, *Trichomonasvirus* and *Totivirus* in the family *Totiviridae*, and it showed the closet homology with PvRV and NoVV1 amongst all the known victoriviruses (Figure 2A). Similarly, aligned CP sequences also showed AalVV1 in a separate cluster with all other victoriviruses (Figure 2B). A comprehensive up-to-date table has been arranged with all the information of the victoriviruses reported so far indicating the name of the virus, size of dsRNA, encoded protein regions, host, GenBank accession numbers, and the presence of conserved sequences in the 5′-UTR (Table S2).

Based on the genomic organization, global amino acid sequence similarities of CP and RdRp, and the phylogeny, AalVV1 appears to represent the genome of a novel victorivirus, based on the demarcation criteria for mycoviruses in this genus. This study represents the first report of the full-length genome sequence of a victorivirus infecting *A. alternata*.

### 3.2. Morphology and Protein Components of Purified AalVV1 Particles

**Figure 3:**
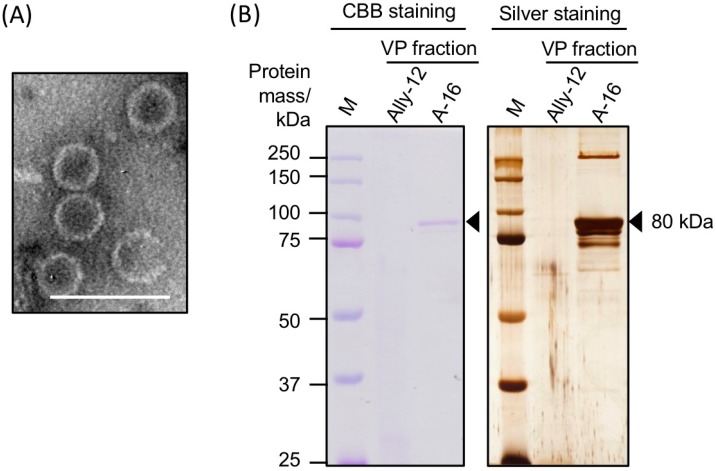
Components of purified virus particles of AalVV1. (**A**) Transmission electron micrograph of negatively stained particles of AalVV1. The white scale bar denotes 100 nm. (**B**) Structural proteins included in the VP fraction of *A. alternata* strain Ally-12 and A-16. Proteins were denatured in modified Laemmli’s sample buffer containing 0.6% 2-mercaptoethanol and electrophoresed in 10% polyacrylamide gels. The gels were stained with Coomassie Brilliant Blue R-250 (CBB) or silver nitrate. The lane M shows approximate protein molecular size with the Precision Plus Protein Dual Color Standards (Bio-Rad Laboratories, Inc., Hercules, California, USA). The black arrows are pointed to the major 80 kDa protein band subjected to LC-MS/MS analysis.

To obtain purified AalVV1 particles, crude extracts of strain A-16 were subjected to CsCl density-gradient centrifugation. The virus-free strain Ally-12 was also subjected to the experiment as a negative control. In the virus-particle (VP) fraction where the AalVV1 dsRNA was enriched, nonenveloped spherical particles of ~40 nm diameter were observed with transmission electron microscope (Figure 3A). Protein components of the VP fraction were detected by SDS-PAGE followed with CBB and silver staining. In the VP fraction of the A-16 strain, a major band corresponding to approximately 80 kDa in size was detected by both staining methods (Figure 3B). In addition, some minor bands sized larger than 250 kDa and smaller than 80 kDa were detected by silver staining of the VP fraction of A-16 (Figure 3B). These bands were undetectable for the samples of a virus-free strain (Ally-12) (Figure 3B). No protein bands in an estimated size of a potential CP-RdRp fusion protein (171 kDa) were observed (Figure 3B).

**Figure 4:**
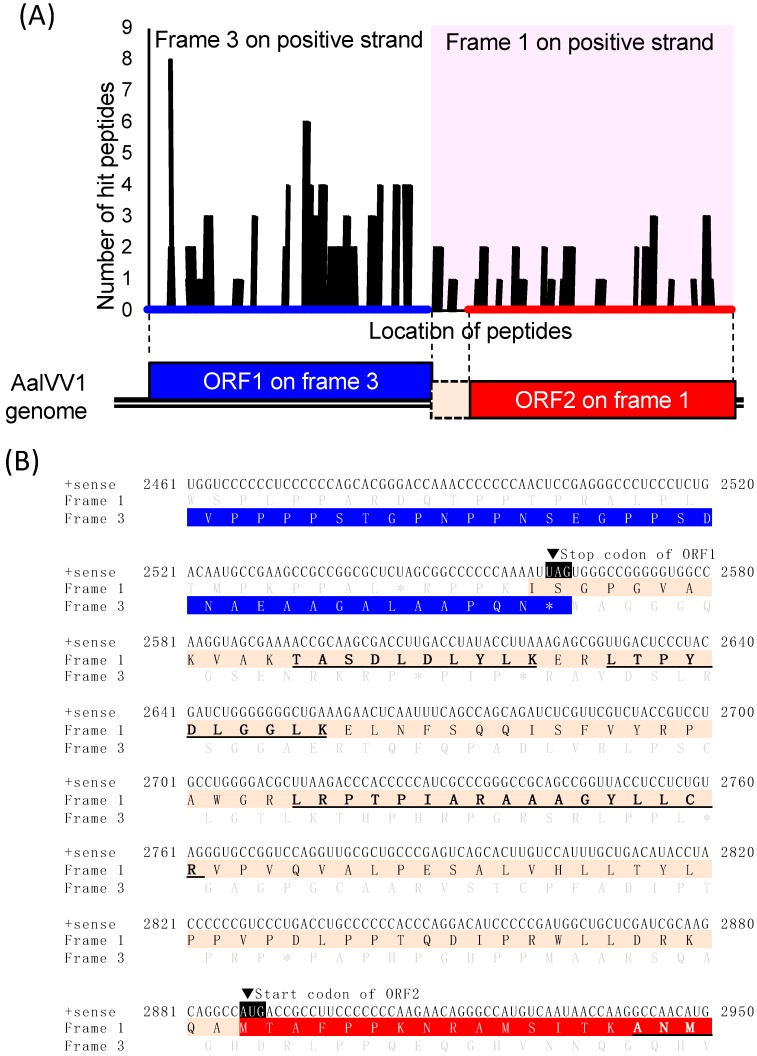
Liquid chromatography–tandem mass spectrometry (LC-MS/MS) analysis of the major 80 kDa protein band of AalVV1. (**A**) The map of peptides obtained by LC-MS/MS analysis of the 80 kDa protein band of AalVV1. The peptides mapped are shown as the black bars on the putative amino acid sequence of frame 3 (267–2564 nt, balck bars in the cyan background) and frame 1 (2560–5043 nt, balck bars in the magenta background) or bridged region between ORF1 and ORF2 (frame 1) on the positive strand of the AalVV1 genome. The vertical axis shows the number of the peptides hit on the indicated positions on the amino acid sequence. The lower diagram represents the AalVV1 genome organization (see Figure 1 legend). The raw data of the LC-MS/MS analysis is shown in Figure S2. (**B**) The nucleic acid sequence (+ sense RNA) and the amino acid sequence (frame +1 and frame +3) of the bridged region between ORF1 and ORF2 of AalVV1 genome. The blue colored letters indicate the predicted amino acid sequence of ORF1, the red colored letters indicate the predicted amino acid sequence of ORF2, and the beige colored letters indicate the predicted amino acid sequence encoded by the bridged sequence of frame 1 between ORF1 and ORF2. The peptides mapped on the bridged region were indicated in bold letters with underline. The stop codon of ORF1 and the start codon of ORF2 are pointed to with the black arrow heads.

Sequences of peptide fragments obtained after in-gel tryptic digestion of the major 80 kDa protein band of A-16 was predicted by liquid chromatography/tandem mass spectrometry (LC-MS/MS). As a result, the band contained the peptides derived from both ORF1 and ORF2 of the AalVV1 genome (Figure 4 and Figure S2). Furthermore, the band also included the peptides translated from frame 1, the same frame as ORF2, of the region between two ORFs (Figure 4A and Figure S2). Note that such peptide sequences are underlined in Figure 4B. A large portion of peptide fragments detected by peptide mass fingerprinting (PMF) were derived from CP-encoding ORF1, while a small but significant portion, approximately one fourth of the former, was derived from RdRp-encoding ORF2 (Figure S2). Considering the estimated protein size of each ORF (Figure 1D), the major 80 kDa band likely contained at least two proteins translated from each ORF. It remains to be elucidated how RdRp and the interval peptides downstream of CP are translated. In any case, purified AalVV1 particles were composed of a major CP and RdRp that were encoded in ORF1 and ORF2, respectively.

### 3.3. Asymptomatic Infection of A. alternata by AalVV1

In order to determine the effect of the virus on the host, attempts were made to create virus-free isogenic line and for that purpose, treatment of a translation inhibition drug cycloheximide, which has been previously used to cure fungus of a mycovirus infection [50], along with the single spore isolation technique was done. *A. alternata* Pakistani A-16 strain was grown on PDA plates amended with different concentrations of cycloheximide (10, 50 and 100 mM) and then single spore isolation was done from the mycelial plugs. However, our attempts to cure A-16 were unsuccessful. Thus, we took a protoplast fusion approach which was used for different viruses.

**Figure 5:**
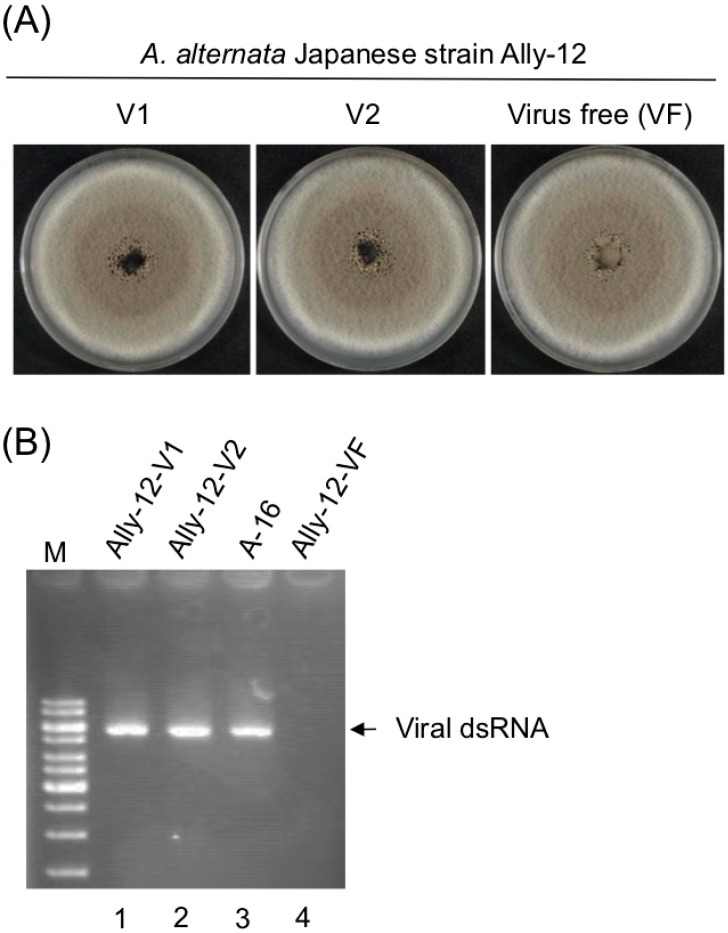
Asymptomatic infection of a Japanese strain of *Alternaria alternata* by AalVV1. (**A**) Colony morphology of a virus free and AlaVV1-infected substrains derived from a Japanese *A. alternata* strain Ally-12. Protoplasts of the virus-free Ally-12 were fused with those of the AalVV1-infected a Pakistani strain of *A. alternata* (A-16). Virus-infected recipient strains with the Ally-12 genetic background were obtained as described in the Materials and Methods section. Virus-free (VF) and virus-infected Ally-12 (V1 and V2) were cultured for five days and photographed. (**B**) Confirmation of AalVV1 infection of Ally-12. DsRNA was isolated from Ally12-V1 (lane 1), Ally-12-V2 (lane 2), A-16 (lane 3), and Ally-12-VF (lane 4), and electrophoresed in agarose gel and stained by EtBr. The 1-kb DNA ladder was used as size standards.

First, a virus-free Japanese strain (Ally-12) of *A. alternata* was transformed with a HygR gene cassette as described by Shahi et al. [46] and used as a primary recipient. After protoplast fusion and hygromycin selection, we conducted several rounds of anastomosis using the untransformed Ally-12 strain (hygromycin B-susceptible, HygS) as a secondary recipient. The virus AalVV1 was finally moved to the original Ally-12 strain (HygS) and compared with the isogenic virus-free Ally-12 strain. Consequently, the two strains showed indistinguishable colony phenotype on PDA, indicating no symptoms induced by AalVV1 (Figure 5). Note that the Japanese virus-free strain Ally-12 showed indistinguishable colony morphology to strain A-16 (see Figure 1A).

### 3.4. AalVV1 is Infectious to the Fungus C. parasitica as Particles and Shows Symptomless Infection

Effects of AalVV1 infection on the fungal phenotype was also tested in a model fungus, *C. parasitica*, as an experimental host, which proved useful for studying virus/host and virus/virus interactions [25]. Spheroplasts of the standard virus-free *C. parasitica* strain EP155 and an RNA silencing-deficient strain, ∆*dcl2*, derived from EP155 were transfected by semipurified AalVV1 particles. Infected colonies were readily obtained in both the EP155 and ∆*dcl2* background and showed an unaltered phenotype which was indistinguishable from virus-free EP155 or ∆*dcl2* (Figure S3).

**Figure 6:**
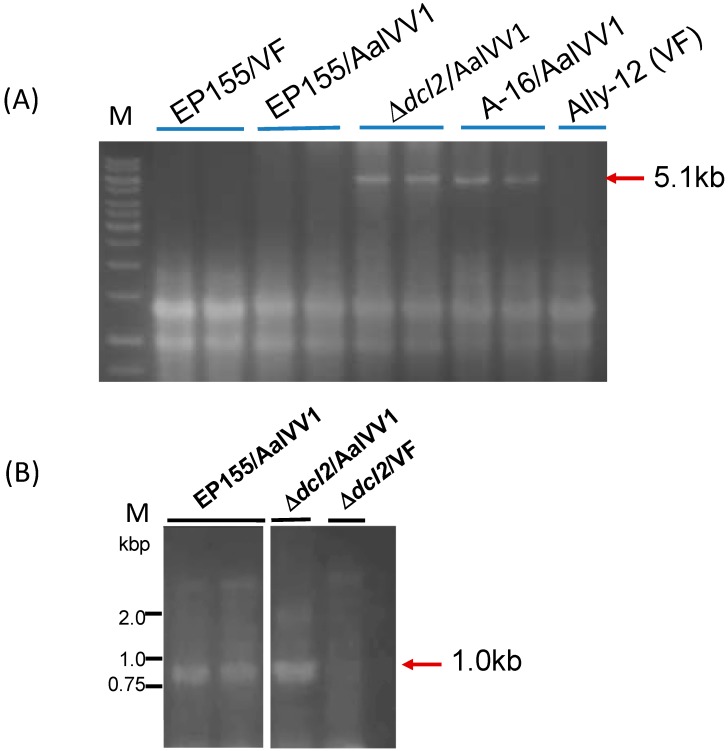
Infection of AalVV1 to a model filamentous fungal host, *Cryphonectria parasitica*. (**A**) Agarose gel electrophoresis of dsRNA from AalVV1-infected *C. parasitica*. Semipurified AalVV1 was transfected into the RNA silencing-competent *C. parasitica* EP155 and -deficient Δ*dcl2* strains. Total RNA extracted from transfectants with either of the genetic backgrounds was electrophoresed in 1.2% agarose gel, and stained by EtBr. AalVV1-infected *A. alternata* strain A-16 and virus-free *A. alternata* strain Ally-12 were analyzed in parallel. The AalVV1 genomic dsRNA of 5.1 kbp is shown by a red arrow. M refers to 1-kb DNA-ladder markers. (**B**) RT-PCR analysis of the EP155 infected by AalVV1. Total RNA fractions were obtained from virus-free Δ*dcl2* and AalVV1-trasnfected EP155 and Δ*dcl2*, and subjected to RT-PCR. Amplified fragments were analyzed in agarose gel electrophoresis. The AalVV1-specific band corresponding to 1 kbp is shown by a red arrow. The migration positions of 1-kb DNA-ladder markers are shown on the left.

Agarose gel electrophoresis of total RNA fractions from transfectants showed AalVV1 accumulation in ∆*dcl2*, comparable to that in *A. alternata* (Figure 6A). A considerable low level of AalVV1 accumulation was observed in wild type EP155. AalVV1 was detectable only by RT-PCR (Figure 6B, see the 1 kbp-specific band).

## 4. Discussion

A number of RNA viruses have been reported from *Alternaria* spp., including dsRNA viruses: an alternavirus [51], a partitivirus [52], botybirnaviruses [43,53,54], chrysoviruses [55], and victoriviruses (a different virus than AalVV1 from *Alternaria arborescens*) [56]; positive-sense (+) single-stranded RNA (ssRNA) viruses: mitoviruses [57,58], a fusarivirus [59], and an endornavirus [60]; and other unidentified viruses [61]. This study describes the characterization of a victorivirus, AalVV1, from *A. alternata*, and contributes to the growing database of viruses of *Alternaria* spp. To our knowledge, this study represents the second report on a fungal virus from the Indian subcontinent next to the paper by Shamsi et al. [43] that reports a thorough characterization of a botybirnavirus (dsRNA virus) of *A. alternata*.

AalVV1 has interesting features similar and dissimilar to previously characterized victoriviruses. For the RdRp translation from bicistronic genome-sized viral mRNA, members of the family *Totiviridae* use −1 frame-shifting (totiviruses, trichomonasviruses, giardiaviruses), −2 frame-shifting (trichomonasvirses), presumable +1 frame-shifting or ribosomal hopping (leishmaniaviruses) and stop/restart strategies (victoriviruses) [10,62] (Figure 2A). The stop/restart translational regulation of victoriviruses was thoroughly explored for HvV190S, the prototype victorivirus [7,8] in which the 38-nt region including the *AUG*A reinitiation facilitator and an H-type pseudoknot structure immediately upstream of the tetra-nucleotide is necessary and sufficient for the stop/restart translation. Similar translational strategies are used in other fungal and animal viruses with (+)ssRNA genomes, such as hypoviruses [23] and caliciviruses [63,64], where 4–20% of ribosomes terminating translation at the stop codon of the upstream ORF reinitiate translation of RdRp. This translational efficiency is similar to that observed for −1 frame-shifting of the prototype totivirus, ScV-L-A [11]. The slippery sites and pseudoknot structures up- and down-stream of the termination codon facilitate −1 frame-shifting.

This study showed AalVV1 to have the sequence features frequently found in −1 frame-shifting and stop/restart mechanisms. AalVV1 has an H-type pseudoknot structure upstream of the ORF1 termination codon (Figure 1) as in the case for HvV190S, and yet lacks the stop/restart facilitator sequence such as *AUG*A or UA*A**UG*. Simultaneously, the virus has a signature-like sequence “C CaA AAU” for possible −1 frame-shifting, slippery sequence conforming to the consensus sequence (X XXY YYZ) at map positions 2555–2561 immediately upstream of the stop codon, UAG, of ORF1, and a predicted stem-loop structure downstream of the stop codon (Figure 1D). Interestingly, the aforementioned H-type pseudoknot structure is retained in AalVV1 immediately upstream of the −1 frame-shifting slippery site (Figure 1D). This study provided some clues, but failed to determine which mechanism is used for the translation of the AalVV1 RdRp ORF. The SDS-PAGE analysis failed to detect the CP-RdRp fusion product expected to be expressed via −1 ribosomal frame-shifting at an expected migration position of 171 kDa. Note that the migration position of a minor band corresponding to >250 kDa is much slower than that expected for the fusion product (171 kDa) (Figure 3B), indicating that the >250 kDa band did not represent the CP-RdRp fusion protein. The PMF indicated that the RdRp peptide fragments were detected in polypeptides of approximately 80 kDa, and that the peptides included amino acid sequences encoded by the ORF1/2 junction region (map positions 2565 to 2886). Namely, it was shown that translation of the −1 frame of ORF2 started upstream of the AUG codon at 2887. These SDS-PAGE and PMF data favor the stop/restart mechanism, but AalVV1 lacks a typical stop/restart facilitator such as *AUG*A and UA*A**UG* around the ORF1/2 junction. If the virus utilizes the −1 frameshift, proteolytic processing or degradation must occur to account for the size (80 kDa) of the detected proteins. If the virus uses termination-coupled reinitiation, there must be an unidentified noncanonical start codon downstream the H-type pseudoknot structure and upstream of the nucleotide sequences (map positions 2593 to 2622) encoding amino acids TASDLDLYLK detected by peptide mass fingerprinting (Figure 4B and Figure S2).

Both the phylogenetic trees with the CP and RdRp sequences showed two unclassified victoriviruses, Nigrospora oryzae victorivirus 1 (NoVV1) and Phomopsis vexans RNA virus (PvRV), to make up a sister clade to the one containing AalVV1 (Figure 2A,B). NoVV1 and PvRV were isolated from fungi phylogenetically distant from *A. alternata*, the original host of AalVV1. This clade does not include another Alternaria victorivirus (Alternaria arborescens victorivirus 1) from *A. arborescens*, the tomato pathotype of *A. alternata* [56]. This is not surprising given that victoriviruses have broad host ranges as in the case of partitviruses [24,26,27]. There might have been horizontal transmission as suggested for other fungal viruses [65,66,67,68]. An additional interesting finding is a possible difference in the translational strategy for ORF2. NoVV1 appears to use the stop/restart mechanism like many other victoriviruses. However, PvRV interestingly lacks a reinitiation facilitator like AalVV1, and ORF2 is situated in the −2 or +1 frame relative to ORF1 (Figure S4).

Victoriviruses are ubiquitous in filamentous fungi [48]. However, only a few victoriviruses have been explored at the molecular level. As aforementioned, the prototypic HvV190S has been utilized for the investigation of viral gene expression [7,8,69]. Biological characterization has been limited to a few victoriviruses largely because inoculation methods had been unavailable until recently. Chiba et al. showed RnVV1 by virion transfection to induce asymptomatic infection in the original fungal host, *R. necatrix* and a model filamentous fungus, *C. parasitica* [26]. Subsequently, Xie et al. succeeded in the transfection of the same fungus with HvV190S as a new experimental host [24]. To our knowledge, HvV190S is the only victorivirus that was shown to induce debilitation and hypovirulence on their original fungal host (*H. victoriae)* [24]. In this study, AalVV1 from a Pakistani *A. alternata* strain (A-16) was shown to cause symptomless infection in wild type strains of a Japanese *A. alternata* (Ally-12) and the RNA silencing-proficient standard strain of *C. parasitica* (EP155). Moreover, unlike RnVV1 and HvV190S [24,26], AalVV1 induced no phenotypic alterations even in an antiviral RNA silencing deficient strain of *C. parastica*, ∆*dcl2*, in spite of the enhanced accumulation of AalVV1 in ∆*dcl2* (Figure 6 and Figure S3), indicating that antiviral RNA silencing operating in *C. parasitica* targets this virus like other viruses [27,32,70,71]. This is a rare phenomenon: while many fungal viruses transfected cause minor symptoms or little in the wild type RNA silencing proficient strain EP155, ∆*dcl2* infected by those viruses show enhanced symptom infection as exemplified by three dsRNA mycoviruses, RnVV1, Rosellinia necatrix partitivirus 2 (RnPV2), and RnPV6 [26,45,72]. A case similar to AalVV1 is a hypovirus (Cryphonectria hypovirus 4) that causes no phenotypic changes in ∆*dcl2* or EP155 [73]. The occasional loss (data not shown) and considerably low accumulation of AalVV1 in EP155 (Figure 6) suggest its greater susceptibility to antiviral RNA silencing. Despite these attributes, this study clearly showed the infectivity of AalVV1 to *C. parasitica*, belonging to a class, Sordariomycetes, different from another one, Dothideomycetes, which include the original host, *A. alternata*. The previous and current studies suggest broad host ranges of victoriviruses in contrast to the relatively narrow host ranges of two other *C. parasitica* viruses, a chrysovirus, Cryphonectria nitschkei chrysovirus 1 [74] and a mitovirus, Cryphonectria parasitica mitovirus 1 [46].

## Figures and Tables

**Table 1:** BlastP results for Alternaria alternata victorivirus 1 (AalVV1) proteins.

Query/Virus name	QC *	*E*-Value	Identity	Accession
Query: AalVV1 ORF1 (putative capsid protein, CP) **				
Phomopsis vexans RNA virus1	92%	0.0	69.82%	YP_009115491.1
Nigrospora oryzae victorivirus 1	92%	0.0	68.86%	YP_009254735.1
Fusarium poae victorivirus 1	93%	0.0	66.34%	YP_009272904.1
Ustilaginoidea virens RNA virus 3	99%	0.0	64.01%	YP_009004155.1
Colletotrichum caudatum totivirus 1	98%	0.0	62.97%	AZT88630.1
Alternaria arborescens victorivirus 1	92%	0.0	67.37%	YP_009553477.1
Coniothyrium minitans RNA virus	92%	0.0	66.95%	ALM62230.1
Ustilaginoidea virens RNA virus 6	94%	0.0	60.00%	ART91348.1
Magnaporthe oryzae virus 3	94%	0.0	60.22%	YP_009143306.1
Gremmeniella abietina RNA virus L2	93%	0.0	61.53%	YP_044806.1
Query: AalVV1 ORF2 (putative RNA-dependent RNA-polymerase, RdRp) **				
Nigrospora oryzae victorivirus 1	99%	0.0	61.36%	YP_009254736.1
Phomopsis vexans RNA virus	99%	0.0	59.53%	YP_009115492.1
Ustilaginoidea virens RNA virus 3	99%	0.0	56.39%	YP_009004156.1
Ustilaginoidea virens RNA virus 6	99%	0.0	54.86%	ART91349.1
Fusarium poae victorivirus 1	99%	0.0	56.39%	YP_009272905.1
Colletotrichum caudatum totivirus 1	99%	0.0	53.69%	AZT88631.1
Coniothyrium minitans RNA virus	99%	0.0	50.21%	YP_392467.1
Phomopsis longicolla totivirus 1	99%	0.0	50.14%	ALD89108.1
Aspergillus mycovirus 178	99%	0.0	48.68%	ABX79995.1
Magnaporthe oryzae virus 3	99%	0.0	49.58%	YP_009143307.1

*: query coverage; **: selected top ten hits.

## Supplementary Materials

The following are available online at https://www.mdpi.com/1999-4915/11/6/577/s1, Figure S1: Multiple alignments of victorivirus nucleotide and amino acid sequences, Figure S2: The Mascot search result of the LC-MS/MS analysis of the major 80 kDa protein band of AalVV1, Figure S3: Colony morphology of Cryphonectria parasitica infected AalVV1, Figure S4: Predicted secondary structure of the ORF1/ORF2 junction sequences of the two AalVV1 related victoriviruses, Table S1: List of primers used in the confirmation and completion of the AalVV1 sequence, Table S2: Some properties of reported victoriviruses.
