# Supplementary material for: Novel Victorivirus from a Pakistani Isolate of Alternaria alternata Lacking a Typical Translational Stop/Restart Sequence Signature
Source: Viruses. 2019 Jun 25;11(6):577. doi: 10.3390/v11060577 (PMC6631646; doi:10.3390/v11060577)

Supplemental Materials

Novel Victorivirus from a Pakistani Isolate of *Alternaria alternata* Lacking a Typical Translational Stop/Restart Sequence Signature

Atif Jamal[1, 2 †], Yukiyo Sato, Sabitree Shahi, Wajeeha Shamsi[1], Hideki Kondo[1], and Nobuhiro Suzuki[1†]

[1]Institute of Plant Science and Resources, Okayama University, Kurashiki, Okayama 710-0046, and [2]Crop Diseases Research Institute, National Agricultural Research Centre, Park Road, Islamabad, 45500 Pakistan.

†Correspondence may be sent to:
Dr. Atif Jamal
Crop Diseases Research Institute
National Agricultural Research Centre
Park Road, Islamabad, 45500 Pakistan
Telephone: +923018544434
E-mail: atif75j@gmail.com

or Dr. Nobuhiro Suzuki
IPSR, Okayama University
Chuou 2-20-1, Kurashiki, JAPAN
Telephone: 81-86-434-1230
FAX: 81-86-434-1232
E-mail: nsuzuki@okayama-u.ac.jp

Supplementary Table S1

Supplementary Figures S1 to S4

Supplementary Table S1 Primer list

Figure S1. Multiple alignments of victorivirus nucleotide and amino acid sequences. A. Presence of Octanucleotide sequence along with an extra seven conserved nucleotides (5′-AGGGUUCC**GUUGAUC**-3′, bold letters) in the 5'UTR of AalVV1 and several other victoriviruses. The numbers in the parenthesis indicate the start of Octanucleotide sequence in the 5'UTR of the respective viruses. **B**. Comparison of the eight conserved motifs of RNA-dependent RNA polymerase of AalVV1 with those of other victoriviruses **C**. Comparison of C-terminal region of AalVV1 with prototype victorivirus HVv190S showing a Ala/Gly/Pro-rich region.

Figure S2. The Mascot search result of the LC-MS/MS analysis of the major 80 kDa protein band of AalVV1. The peptides predicted by LC-MS/MS analysis were mapped onto the deduced amino acid sequence of AalVV1 under the assumption that ribosomes were slipped on repeated A sequences followed with the stop codon of the ORF1. The light cyan and the light magenta indicate the amino acid sequence of frame +3 and frame +1, respectively, on the positive strands of AalVV1 genome.

Figure S3. Colony morphology of *Cryphonectria parasitica* infected AalVV1. Protoplasts of two strains of *C. parasitica*, the standard RNA silencing-proficient EP155 and RNA silencing-deficient strain Ddcl2, were transfected by partially purified virions of AalVV1. Obtained transfectants and virus-free counterparts were grown on PDA for one week, and photographed.

Figure S4. Predicted secondary structure of the ORF1/ORF2 junction sequences of the two AalVV1 related victoriviruses. Pseudoknot structures were predicted by DotKnot program [Sperschneider and Datta, 2010] and schematically represented for Phomopsis vexans RNA virus (PvRV, accession number KP090346) and Nigrosopra oryzae victorivirus 1 (NoVV1, accession number KT428155). The ORF1 termination codon and the ORF2 AUG codon are denoted in blue and red, while the upstream in-frame ORF2 stop codons are shown in bold. PvRV could utilize the -2 /+1 frameshift and the possible slippery site (blue bracket) is shown. A predicted RNA pseudoknot structure is depicted near the 3′ end of the ORF1, and the nucleotide positions are indicated (2511–2538), whereas NoVV1 could utilize the terminitation-reinitiation strategy and the position of overlapping start codon of the ORF2 and the stop codon of the ORF1 in the tetranucleotide sequence *A<u>UG</u>A* (map position 2554, highlighted red/blue) as indicated.

Sperschneider, J.; Datta, A., DotKnot: pseudoknot prediction using the probability dot plot under a refined energy model. *Nucleic Acids Res* **2010,** 38, (7), e103.

Table S1. List of primers used in the confirmation and completion of the AalVV1 sequence.

Primer name	Oligonucleotide sequence (5'-3')	Position	Usage
AJ- 19F (1)	CCGATGGCCGATGAGAACAGG	2244-2264	RT-PCR
AJ- 19R (1)	TCTCCAGCAATCCCGCGCTAC	2973-2953	RT-PCR
AJ- 19F (2)	CGCATCCCTTCAAGTTCCGGG	2035-2055	RT-PCR
AJ- 19R (2)	AGATCGTAGGGAGTCAACCGC	2645-2625	RT-PCR
AJ- 19 (E1)	GTAGCGTCGGTATTGGTCGTC	374-353	RLM-RACE
AJ- 19 (E1)	GCACCGTCGGGTGTGCTCACC	4812-4792	RLM-RACE
AJ- 19 (E2)	TGAGTGTAGGCCATCGCCTCG	493-473	RLM-RACE
A2- 19 (E2)	GATGGTCATGTCTAGTTGGTC	4662-4681	RLM-RACE
LIG-For primer	CCGCTCTAGAACTAGTTGGATC		RLM-RACE
LIG-Rev [5'-phosphorylated; 3'-amino-linked]	GATCCAACTAGTTCTAGAGCGG		RLM-RACE
ITS1	TCCGTAGGTGAACCTGCGG		For rDNA amplifiction
ITS2	TCCTCCGCTTATTGATATGC		For rDNA amplifiction

Table S2. Some properties of reported victoriviruses

SNo	Virus name	Abbreviations	Accession	Genome length (nt)	CP coding region	RdRp coding region	Host	Conserved octanucleotide sequence in 5'UTR	Reference
1	Aspergillus foetidus slow virus 1	AfSV1	NC_038928	5194	374-2599	2599-5188	Fungi	**AGGGTTCCGTTGATC**	Kozlakidis *et al*., 2013
2	Beauveria bassiana victorivirus 1	BbVV-1	NC_038929	5288	444-2672	2672-5176	Fungi	**AGGGTTCCGTTGATC**	Herrero *et al*., 2012
3	Beauveria bassiana victorivirus NZL/1980	BbVV-NZL/ 1980	NC_024151	5327	459-2735	2735-5266	Fungi	**AGGGTTCCGTTGATC**	Khalifa. (unpublished)
4	Botryosphaeria dothidea victorivirus 1	BdV1	NC_025214	5322	512-2743	2743-5265	Fungi	----------	Zhai et al. (unpublished)
5	Chalara elegans RNA Virus 1	CeRV1	NC_005883	5310	329-2641	2619-4067	Fungi	----------	Park *et al*., 2005
6	Coniothyrium minitans RNA virus	CmRV	NC_007523	4975	62-2389	2386-4875	Fungi	**AGGGTTCCGTTGATC (start of UTR)**	Cheng *et al*., 2003
7	Eimeria tenella RNA virus 1	EtV 1	NC_026140	6006	350-2716	2713-5928	Protozoa	----------	Wu *et al*., 2016
8	Epichloe festucae virus 1	EfV1	NC_038930	5109	271-2571	2568-5051	Fungi	**AGGGTTCC**	Romo *et al*., 2007
9	Fusarium poae victorivirus 1	FpV 1	NC_030867	5124	164-2572	2572-5055	Fungi	**AGGGTTCC (in ORF1)**	Osaki *et al*., 2016
10	Gremmeniella abietina RNA virus L1	GaRV-L1	NC_003876	5133	276-2603	2603-5080	Fungi	**AGGGTTCC**	Tuomivirta and Hantula, 2003
11	Helicobasidium mompatotivirus 1-17	HmTV1-1-17	NC_005074	5207	200-2566	2563-5100	Fungi	----------	Nomura *et al*., 2003
12	Helminthosporium victoriae virus 190S	HvV190S	NC_003607	5179	290-2608	2605-5112	Fungi	----------	Huang and Ghabrial, 1996
13	Magnaporthe oryzae virus 1	MoV1	NC_006367	5359	575-2815	2818-5316	Fungi	----------	Yokoi *et al*., 2007
14	Magnaporthe oryzae virus 2	MoV2	NC_010246	5139	275-2641	2638-5130	Fungi	**AGGGTTCC**	Maejima *et al*., 2008
15	Magnaporthe oryzae virus 3	MoV3	NC_027209	5181	283-2634	2631-5108	Fungi	**AGGGTTCC**	Tang *et al*., 2015
16	Nigrospora oryzae victorivirus 1	NoRV1	NC_030224	5100	236-2554	2554-5037	Fungi	**AGGGTTCCGTTGATC**	Zhong *et al*., 2016
17	Phomopsis vexans RNA virus	PvRV1	NC_026135	5076	243-2543	2642-5026	Fungi	**AGGGTTCCGTTGATC**	Zhang *et al*., 2015
18	Rosellinia necatrix victorivirus 1	RnVV1	NC_021565	5329	373-2664	2664-5192	Fungi	----------	Chiba *et al*., 2013
19	Sclerotinia nivalis victorivirus 1	SnVV1	NC_030392	5162	286-2583	2580-5081	Fungi	**AGGGTTCC**	Wu and Li. (unpublished)
20	Sphaeropsis sapinea RNA virus 1	SsRV1	NC_001963	5163	80-2581	2574-5090	Fungi	**AGGGTTCCGTTGATC (in ORF1)**	Preisig *et al*., 1998
21	Sphaeropsis sapinea RNA virus 2	SsRV2	NC_001964	5202	296-2665	2658-5135	Fungi	**AGGGTTCC**	Preisig *et al*., 1998
22	Tolypocladium cylindrosporum	TcV1	NC_014823	5196	327-2603	2604-5126	Fungi	----------	Herrero and

	virus 1								Zabalgogeazcoa, 2011
23	Ustilaginoidea virens RNA virus 1	UvRV1	NC_020997	5142	274-2604	2904-5075	Fungi	----------	Zhong *et al.*, 2014
24	Ustilaginoidea virens RNA virus 3	UvRV3	NC_023547	5075	259-2532	2532-5021	Fungi	**AGGGTTCCGTTGATC**	Zhong *et al.*, 2014
25	Ustilaginoidea virens RNA virus 5	UvRV5	NC_028477	5221	314-2584	2584-5166	Fungi	**AGGGTTCCGTTGATC**	Zhong *et al.* (unpublished)
26	Ustilaginoidea virens RNA virus L	UvRVL	NC_025366	5654	421-2661	2661-5144	Fungi	**AGGGTTCC**	Jiang et al., 2015
27	Alternaria arborescens victorivirus 1	AaVV1	NC_040793	5206	327-2657	2654-5143	Fungi	**AGGGTTCCGTTGATC**	Komatsu *et al.*, 2016
28	Botryosphaeria dothidea victorivirus 2	BdVV2	MH301088	5,090	284-2505	2512-5019	Fungi	**AGGGTTCCGTTGATC**	Yang *et al.*, 2019
29	Fusarium asiaticum victorivirus 1	FaVV1	NC_040653	5281	378-2657	2657-5185	Fungi	-----------	Li *et al.*, 2019
30	Nigrospora oryzae victorivirus 2	NoRV2	MH823900	5166	327-2597	2597-5095	Fungi	----------	Liu *et al.*, 2019
31	Ustilaginoidea virens RNA virus M	UvRVM	NC_025367	2714	731-2239		Fungi	--------	Jiang *et al.*, 2014

Figure S1

A

```
EfV1       [215]  GAGGAGGGUUCCUAAGAUCCCAAACUUUACGAAUAUC----------CCAAC
GaRV-L1    [215]  GGCGAGGGUUCCGAAGACCCCU-AAA------------CUACA---ACAAU
MoV2       [207]  GAGGAGGGUUCCGUUGAUACCA-ACG-AUUUAACAACUAGUAAC---ACGAU
MoV3       [216]  AAGGAGGGUUCCGAAGAUCUCU-ACAUAAGAAACGAUUGUUACA---GUAAC
SnVV1      [194]  GAGUAGGGUUCCGUUGAUCCCACACUUGUUAGAUUGACGAAAUAC-AGUAAC
SsRV2      [229]  GAGGAGGGUUCCGAAGAUCCAA-ACC-AUUACACACCUGCAACU---CUGAU
UvRVL      [332]  GAGGAGGGUUCCGUUGAUCCUG-GCCUACCAGUGUUCUAACGUACUAGUGCU
                  ..  ********   **. .   .                       .. .
```

```
AalVV1     [204]--------------CUGGGUCCGUAGAGGAGGGUUCCGUUGAUCCUACUACCAG-
AfSV1      [300]------------------------AGGGAGGGUUCCGUUGAUCUCAGCCUAGAC
BbVV-1     [352]UGGCUUCACAGGAGAUAAGUGACUUGGGGAGGGUUCCGUUGAUCCAAGCCA----
BbVV-NZL/  [382]UAGAAUCGGCCGUUCUGUGUAUGUGGGGGAGGGUUCCGUUGAUCCCACCUACGCU
CmRV       [1]------------------------------AGGGUUCCGUUGAUCCACCUGCUCAA
NoRV1      [172]---------------UUGUUCCGGCGGGGAGGGUUCCGUUGAUCCGACCUUCAAC
PvRV1      [182]----------------------GCGGGGAGGGUUCCGUUGAUCCGACCUGCACC
UvRV3      [196]------------AAGUAUGUUUCGGGAGUAGGGUUCCGUUGAUCCCCGU------
UvRV5      [168]--------------CACAGGCGGAGGAGGAGGGUUCCGUUGAUCCUCGAGUUUGU
                                             ***************
```

B

```
                 Motif I              Motif II              Motif III
AalVV1   [103] GSLLVETDTLQGR[63]SRWNWSVNGSHSA [46] KLECGKTRAIYACDTRSY[46]
NoVv1    [211] GALLVETDTLLGR[64]SRWSWAVNGSHSA [46] KPECGKIRAIYACDTINY[46]
PvRV     [178] GALLVETDTLQGR[64]SRWSWAVNGSHSS [46] KLECGKTRAIYACDTVSY[46]
UvRV3    [212] GAMMVETDTLAGR[65]SRWRWAVNGSHSA [46] KLEHGKTRTILACDTRSY[46]
UvRV6    [213] GAMMVEGDTLAGR[65]SRWRWAVNGSHSA [46] KLEHGKTRTILACDTRSY[46]
FpV1     [211] GSLLVECEVLQGR[64]SRWVWAVNGSQSS [46] KLENGKTRAIFACDTRHY[46]
HVv190S  [232] GALFVEANTLQGR[58]SRWLWCVNGSQNA [42] KLENGKDRAIFACDTRSY[46]
               *:::** :.* **    *** *.****:.:       * * ** *:* ****  *
```

```
               Motif IV             Motif V                        Motif VI
AalVV1       MLDYDDFNSHHT [45] GTLMSGHRGTTYFNSVLNMAYLMCVLGEDYILARPSLHVGDDVY
NoVv1        MLDYDDFNSHHT [45] GTLMSGHRCTTYVNSVLNMAYLMVVLGEDWVMERQSLHVGDDVY
PvRV         MLDYTDFNSHHT [45] GTLMSGHRCTTYINSVLNMAYLMVVLGEDFVLERQSLHVGDDVY
UvRV3        MLDYDDFNSHHS [45] GTLMSGHRCTTYINSVLNMAYLMVVLGDDFVMERPTLHVGDDVF
UvRV6        MLDYDDFNSHHS [45] GTLMSGHRCTTYINSVLNMAYLMVVLGDDFVMDRPTLHVGDDVF
FpV1         MLDYDDFNSQHT [45] GTLMSGHRATTFINSVLNKAYLDVVLGEGWLDTRRSVHVGDDIY
HVv190S      MLDYDNFNSQHS [44] GTLMSGHRATTFTNSVLNAAYICYAVGIPAFKRMISLHAGDDVY
             **** :***:*:      ******** **: ***** **:  .:*   .    ::*.***::
```

```
                     Motif VII                 Motif VIII
AalVV1    [21] RMNPTKQSVGHVSTEFLRVASDARDSYGYLARAVATTVAGNWYT [250]
NoVv1     [21] RMNRRKQSVGHVSTEFLRVASDARDSYGYLARAAANLIAGNWYS [250]
PvRV      [21] RMNKSKQSVGHVGTELTRVASNKRDSFGYLARATANLIAGNWYS [250]
UvRV3     [21] RMNRSKQSVGHVATEFLRVSSRARDSYGYLCRAISSCVSGNWVS [250]
UvRV6     [21] RMNRSKQSVGHVSTEFLRVSSQGRVSMGYLCRAIASTVSGNWVT [250]
FpV1      [21] RMNPTKQSVGHVSTEFLRLATAGRDTYGYVARSIASLISGNWVS [250]
HVv190S   [21] RMNPTKQSIGYTGAEFLRLGINKSYAIGYLCRAIASLVSGSWTS [248]
               ***  ***:*:..:*: *:.     : **:.*: :. ::*.* :
```

C

```
HVv190S   [696] LRAPPFPRQQGAL-GGGGNVPLPPAPGAAPPPPPGPPNGPPAGPPPSDDGSSNPAAPVPTA
AalVv1    [704] LRGAPLPRVGGGQLGGAVNPPPPPQGGLVPPPPSTGPNPPNSEGPPSDNAEAAGALAAPQN
                **. *:**  *.  **. * * **  * .****   ** * :  ****:..:  *   .*
```

Figure S2

{MATRIX SCIENCE} **Mascot Search Results**

Protein View

Match to: **AalVV1_frameshift** Score: **2456**

Found in search of AalVV1_80kDa.mgf

Nominal mass (M_r): **171617**; Calculated pI value: **6.96**
NCBI BLAST search of AalVV1 frameshift against nr
Unformatted sequence string for pasting into other applications

Fixed modifications: Carbamidomethyl (C)
Cleavage by Trypsin: cuts C-term side of KR unless next residue is P
Sequence Coverage: **36%**

Matched peptides shown in **Bold Red**

1 MASTVNFQIA ANAMLTGTLS GVSGGLLQAD DQYRRYRAGL SVGSFEHGSY
51 THSR**RSIFYE** **VGRR**YGRLVE AMAYTHGNED AVEIDASVPI NPAQAANFEG
101 WAR**RFSNFSP** **QWVMMDLAGV** **AER**LAK**GVAA** **QSVYGGVSCG** **HLR**GGAGIR**I**
151 **VALGTLDSPQ** **TASNNSVFIP** **R**TVDTVGNDH VFAVLAAAAN GEGATVTTDV
201 VRLDAATNQP IVPNVDGYAF ATACVEALR**V** **LGANMEESGA** **GDMFAYAVTR**
251 GIHSIVSVVA HTDEGGWLRR VLRGSSFR**VP** **YGGINQALR**D YPGLPPLAGH
301 FTSAVSAWVD AIALKTAAAV AHCDPCTIAT GGSYPTVFTS ATGAVSAPGT
351 DETDAGDADS RTIGR**QISSD** **SGRFAPLFVR** ALTGIFGLNT ISGVAEAFHC
401 TAAGQELLES TDRHLRHK**TV** **APYFWIEPTS** **LIPRNAFGTE** **AERAGFGSIT**
451 **SVGLESEMPA** **FERVRELDR**G R**NANFSTIAF** **K**MRTAR**TSGL** **ISAYAGKPAD**
501 **LSGFRLYQFD** **EDSIILPGDQ** **GPTAGTVAAK** **HAAADPITSY** **LWR**RGQSPIP
551 **APAEFINTQS** **MYAAK**YKIVD WDDDFDATLG DLPEAWELET HPFKFR**VTSP**
601 **TAIANGGSNA** **GDNMAK**RARS **RAAIALAQAT** **IR**NRGLGDAS SPVLSVSNVP
651 PTFDDTPAAP MADENR**VEHH** **SDPGPTQRFG** **LGNDPSTAPS** **VRGPAALPIL**
701 **HQQPLRGAPL** **PR**VGGGQLGG AVNPPPPPQG GLVPPPPSTG PNPPNSEGPP
751 SDNAEAAGAL AAPQISGPGV AKVAK**TASDL** **DLYLKE**R**LTP** **YDLGGLK**ELN
801 FSQQISFVYR PAWGR**LRPTP** **IARAAAGYLL** **CR**VPVQVALP ESALVHLLTY
851 LPPVPDLPPT QDIPRWLLDR KQAMTAFPPK NRAMSITK**AN** **MFLDEVAR**DC
901 WR**VWPGLLQQ** **ASPYLAR**LRD MKATHDQATA YLLYSVALSH HTIQAFR**WAF**
951 **YALSNPK**GAK EVSNFLKAVG GNASSFGSLL VETDTLQGR**D** **TPGTNLAEDA**
1001 **K**KR**TNLGAIK** **NDMLAEFDDD** **LLR**SAVRRVI DLELAHPGEE GYQFPTLADH
1051 WSSRWNWSVN GSHSALVGR**E** **IGSLPYPK**ER ISKMHRR**AWL** **ECVEEDPR**VG
1101 WDGHTYVSAN PKLECGKTR**A** **IYACDTRSYL** **AFEHLLATVE** **R**NWRGSR**VIL**
1151 **NPGR**GGHIGM AERVARNRRR CGISLMLDYD DFNSHHTTRA MQIVIEETCS
1201 ATSYPPDLAA PLIASLGK**QD** **IYLDGK**FVGR **SAGTLMSGHR** GTTYFNSVLN
1251 MAYLMCVLGE DYILARPSLH VGDDVYMGAT TYTEVGHIVE TVMASRLRMN
1301 PTKQSVGHVS TEFLRVASDA R**DSYGYLAR**A VATTVAGNWY TDR**VLNPFEA**
1351 **LTTMVTAAR**T LANRAR**SNLV** **PLLLGSAVK**R VLGPDSPDDT MVDEILCGGL
1401 AINNGPVFSS GGTLR**AVTVE** **PTIK**SRDNAG YQELPCLSSN EFLSKCASPL
1451 ETTILSEAGI SVKKQMVMSS WSKSVNFR**DA** **DLLGLR**FGTP TSHPAIGSVS
1501 AESLIK**TRAP** **SGVLTKYPLL** **VLAKGRLPET** **ALR**VAVAAAG GNPNAPDIML
1551 EAWGEYKHGC IVNTVLSYSD AAALSVRTAC SVLTSTRRCY V

ORF1 on frame 3 (stop codon and N ahead of it were removed)

Bridged sequence on frame 1

ORF2 on frame 1

Show predicted peptides also

Sort Peptides By ◉ Residue Number ○ Increasing Mass ○ Decreasing Mass

Start - End	Observed	Mr(expt)	Mr(calc)	ppm	Miss	Sequence	
55 - 63	563.8062	1125.5979	1125.5931	4	1	R.RSIFYEVGR.R	(Ions score 35)
55 - 63	563.8062	1125.5979	1125.5931	4	1	R.RSIFYEVGR.R	(Ions score 38)
56 - 63	485.7548	969.4950	969.4920	3	0	R.SIFYEVGR.R	(Ions score 31)
56 - 63	485.7548	969.4950	969.4920	3	0	R.SIFYEVGR.R	(Ions score 25)
56 - 63	485.7548	969.4950	969.4920	3	0	R.SIFYEVGR.R	(Ions score 22)
56 - 63	485.7548	969.4950	969.4920	3	0	R.SIFYEVGR.R	(Ions score 10)
56 - 64	563.8062	1125.5979	1125.5931	4	1	R.SIFYEVGRR.Y	(Ions score 20)
56 - 64	563.8062	1125.5979	1125.5931	4	1	R.SIFYEVGRR.Y	(Ions score 10)
104 - 123	781.0576	2340.1510	2340.1143	16	1	R.RFSNFSPQWVMMDLAGVAER.L	(Ions score 2)
105 - 123	728.9977	2183.9714	2184.0132	-19	0	R.FSNFSPQWVMMDLAGVAER.L	(Ions score 10)
127 - 143	859.4282	1716.8419	1716.8366	3	0	K.GVAAQSVYGGVSCGHLR.G	(Ions score 45)
150 - 171	1150.6072	2299.1998	2299.2172	-8	0	R.IVALGTLDSPQTASNNSVFIPR.T	(Ions score 93)
150 - 171	1150.6210	2299.2275	2299.2172	4	0	R.IVALGTLDSPQTASNNSVFIPR.T	(Ions score 117)
150 - 171	1150.6210	2299.2275	2299.2172	4	0	R.IVALGTLDSPQTASNNSVFIPR.T	(Ions score 83)
230 - 250	1095.0037	2187.9929	2187.9929	0	0	R.VLGANMEESGAGDMFAYAVTR.G	(Ions score 85)
279 - 289	594.3322	1186.6499	1186.6458	3	0	R.VPYGGINQALR.D	(Ions score 59)
279 - 289	594.3322	1186.6499	1186.6458	3	0	R.VPYGGINQALR.D	(Ions score 59)
279 - 289	594.3322	1186.6499	1186.6458	3	0	R.VPYGGINQALR.D	(Ions score 47)
279 - 289	594.3322	1186.6499	1186.6458	3	0	R.VPYGGINQALR.D	(Ions score 4)
366 - 373	425.2060	848.3975	848.3988	-2	0	R.QISSDSGR.F	(Ions score 28)
366 - 373	425.2060	848.3975	848.3988	-2	0	R.QISSDSGR.F	(Ions score 31)
374 - 380	425.2543	848.4941	848.4909	4	0	R.FAPLFVR.A	(Ions score 33)
374 - 380	425.2543	848.4941	848.4909	4	0	R.FAPLFVR.A	(Ions score 28)
374 - 380	425.2543	848.4941	848.4909	4	0	R.FAPLFVR.A	(Ions score 26)
374 - 380	425.2543	848.4941	848.4909	4	0	R.FAPLFVR.A	(Ions score 26)
419 - 434	945.5130	1889.0115	1889.0087	1	0	K.TVAPYFWIEPTSLIPR.N	(Ions score 15)
419 - 434	945.5130	1889.0115	1889.0087	1	0	K.TVAPYFWIEPTSLIPR.N	(Ions score 21)
419 - 434	945.5130	1889.0115	1889.0087	1	0	K.TVAPYFWIEPTSLIPR.N	(Ions score 57)
419 - 434	630.6789	1889.0148	1889.0087	3	0	K.TVAPYFWIEPTSLIPR.N	(Ions score 8)
419 - 434	630.6789	1889.0148	1889.0087	3	0	K.TVAPYFWIEPTSLIPR.N	(Ions score 36)
419 - 434	630.6789	1889.0148	1889.0087	3	0	K.TVAPYFWIEPTSLIPR.N	(Ions score 41)
435 - 443	497.7345	993.4544	993.4516	3	0	R.NAFGTEAER.A	(Ions score 44)
435 - 443	497.7345	993.4544	993.4516	3	0	R.NAFGTEAER.A	(Ions score 46)
435 - 443	497.7345	993.4544	993.4516	3	0	R.NAFGTEAER.A	(Ions score 37)
435 - 443	497.7345	993.4544	993.4516	3	0	R.NAFGTEAER.A	(Ions score 37)
444 - 463	1043.0077	2084.0009	2083.9884	6	0	R.AGFGSITSVGLESEMPAFER.V	(Ions score 39)
444 - 463	1043.0077	2084.0009	2083.9884	6	0	R.AGFGSITSVGLESEMPAFER.V	(Ions score 71)
444 - 463	695.6770	2084.0092	2083.9884	10	0	R.AGFGSITSVGLESEMPAFER.V	(Ions score 27)
464 - 469	394.2262	786.4378	786.4348	4	1	R.VRELDR.G	(Ions score 13)
464 - 469	394.2262	786.4378	786.4348	4	1	R.VRELDR.G	(Ions score 6)
464 - 469	394.2262	786.4378	786.4348	4	1	R.VRELDR.G	(Ions score 5)
464 - 469	394.2262	786.4378	786.4348	4	1	R.VRELDR.G	(Ions score 13)
472 - 481	556.7924	1111.5703	1111.5662	4	0	R.NANFSTIAFK.M	(Ions score 55)
472 - 481	556.7924	1111.5703	1111.5662	4	0	R.NANFSTIAFK.M	(Ions score 52)
472 - 481	556.7924	1111.5703	1111.5662	4	0	R.NANFSTIAFK.M	(Ions score 51)
472 - 481	556.7924	1111.5703	1111.5662	4	0	R.NANFSTIAFK.M	(Ions score 52)
487 - 505	956.0062	1909.9979	1909.9898	4	0	R.TSGLISAYAGKPADLSGFR.L	(Ions score 37)
487 - 505	956.0062	1909.9979	1909.9898	4	0	R.TSGLISAYAGKPADLSGFR.L	(Ions score 47)
506 - 530	1303.6586	2605.3026	2605.2912	4	0	R.LYQFDEDSIILPGDQGPTAGTVAAK.H	(Ions score 22)
506 - 530	1303.6586	2605.3026	2605.2912	4	0	R.LYQFDEDSIILPGDQGPTAGTVAAK.H	(Ions score 57)
531 - 543	750.8861	1499.7577	1499.7521	4	0	K.HAAADPITSYLWR.R	(Ions score 20)
531 - 543	750.8861	1499.7577	1499.7521	4	0	K.HAAADPITSYLWR.R	(Ions score 44)
531 - 543	750.8861	1499.7577	1499.7521	4	0	K.HAAADPITSYLWR.R	(Ions score 49)
545 - 565	1111.0559	2220.0973	2220.0885	4	0	R.GQSPIPAPAEFINTQSMYAAK.Y	(Ions score 66)
545 - 565	1111.0559	2220.0973	2220.0885	4	0	R.GQSPIPAPAEFINTQSMYAAK.Y	(Ions score 52)
597 - 616	938.4505	1874.8865	1874.8792	4	0	R.VTSPTAIANGGSNAGDNMAK.R	(Ions score 20)
597 - 616	938.4505	1874.8865	1874.8792	4	0	R.VTSPTAIANGGSNAGDNMAK.R	(Ions score 106)
597 - 616	938.4505	1874.8865	1874.8792	4	0	R.VTSPTAIANGGSNAGDNMAK.R	(Ions score 86)
622 - 632	549.8369	1097.6592	1097.6557	3	0	R.AAIALAQATIR.N	(Ions score 62)
622 - 632	549.8369	1097.6592	1097.6557	3	0	R.AAIALAQATIR.N	(Ions score 63)
622 - 632	549.8369	1097.6592	1097.6557	3	0	R.AAIALAQATIR.N	(Ions score 59)
622 - 632	549.8369	1097.6592	1097.6557	3	0	R.AAIALAQATIR.N	(Ions score 47)
667 - 678	453.8869	1358.6389	1358.6327	5	0	R.VEHHSDPGPTQR.F	(Ions score 23)
667 - 678	453.8869	1358.6389	1358.6327	5	0	R.VEHHSDPGPTQR.F	(Ions score 4)
667 - 678	453.8869	1358.6389	1358.6327	5	0	R.VEHHSDPGPTQR.F	(Ions score 27)
667 - 678	453.8869	1358.6389	1358.6327	5	0	R.VEHHSDPGPTQR.F	(Ions score 29)
679 - 692	709.3596	1416.7046	1416.6998	3	0	R.FGLGNDPSTAPSVR.G	(Ions score 85)
679 - 692	709.3596	1416.7046	1416.6998	3	0	R.FGLGNDPSTAPSVR.G	(Ions score 81)
679 - 692	709.3596	1416.7046	1416.6998	3	0	R.FGLGNDPSTAPSVR.G	(Ions score 65)
693 - 706	755.9500	1509.8854	1509.8780	5	0	R.GPAALPILHQQPLR.G	(Ions score 31)
693 - 706	755.9500	1509.8854	1509.8780	5	0	R.GPAALPILHQQPLR.G	(Ions score 50)
693 - 706	755.9500	1509.8854	1509.8780	5	0	R.GPAALPILHQQPLR.G	(Ions score 43)
693 - 706	755.9500	1509.8854	1509.8780	5	0	R.GPAALPILHQQPLR.G	(Ions score 45)
707 - 712	305.6880	609.3615	609.3598	3	0	R.GAPLPR.V	(Ions score 32)
707 - 712	305.6880	609.3615	609.3598	3	0	R.GAPLPR.V	(Ions score 22)
707 - 712	305.6880	609.3615	609.3598	3	0	R.GAPLPR.V	(Ions score 22)
707 - 712	305.6880	609.3615	609.3598	3	0	R.GAPLPR.V	(Ions score 22)
776 - 785	569.8033	1137.5921	1137.5917	0	0	K.TASDLDLYLK.E	(Ions score 12)
776 - 785	569.8033	1137.5921	1137.5917	0	0	K.TASDLDLYLK.E	(Ions score 38)
788 - 797	538.8050	1075.5954	1075.5914	4	0	R.LTPYDLGGLK.E	(Ions score 23)
788 - 797	538.8050	1075.5954	1075.5914	4	0	R.LTPYDLGGLK.E	(Ions score 23)
816 - 823	462.2947	922.5748	922.5712	4	0	R.LRPTPIAR.A	(Ions score 2)
824 - 832	497.7607	993.5069	993.5066	0	0	R.AAAGYLLCR.V	(Ions score 1)
889 - 898	583.2910	1164.5675	1164.5597	7	0	K.ANMFLDEVAR.D	(Ions score 28)
903 - 917	849.9750	1697.9355	1697.9253	6	0	R.VWPGLLQQASPYLAR.L	(Ions score 7)
903 - 917	849.9750	1697.9355	1697.9253	6	0	R.VWPGLLQQASPYLAR.L	(Ions score 14)
948 - 957	598.8138	1195.6130	1195.6026	9	0	R.WAFYALSNPK.G	(Ions score 18)
990 - 1001	616.2973	1230.5801	1230.5728	6	0	R.DTPGTNLAEDAK.K	(Ions score 36)
990 - 1001	616.2973	1230.5801	1230.5728	6	0	R.DTPGTNLAEDAK.K	(Ions score 18)
1004 - 1010	358.7212	715.4279	715.4228	7	0	R.TNLGAIK.N	(Ions score 22)
1011 - 1023	783.8613	1565.7081	1565.7032	3	0	K.NDMLAEFDDDLLR.S	(Ions score 10)
1070 - 1078	502.2795	1002.5445	1002.5386	6	0	R.EIGSLPYPK.E	(Ions score 16)
1070 - 1078	502.2795	1002.5445	1002.5386	6	0	R.EIGSLPYPK.E	(Ions score 14)
1088 - 1098	702.3156	1402.6167	1402.6187	-1	0	R.AWLECVEEDPR.V	(Ions score 18)
1088 - 1098	702.3156	1402.6167	1402.6187	-1	0	R.AWLECVEEDPR.V	(Ions score 9)
1120 - 1127	485.2258	968.4370	968.4386	-2	0	R.AIYACDTR.S	(Ions score 17)
1128 - 1141	550.2978	1647.8716	1647.8620	6	0	R.SYLAFEHLLATVER.N	(Ions score 2)
1128 - 1141	824.9469	1647.8792	1647.8620	10	0	R.SYLAFEHLLATVER.N	(Ions score 3)
1148 - 1154	384.7411	767.4677	767.4654	3	0	R.VILNPGR.G	(Ions score 12)
1148 - 1154	384.7411	767.4677	767.4654	3	0	R.VILNPGR.G	(Ions score 26)
1219 - 1226	476.2435	950.4725	950.4709	2	0	K.QDIYLDGK.F	(Ions score 32)
1231 - 1240	508.7512	1015.4878	1015.4869	1	0	R.SAGTLMSGHR.G	(Ions score 10)
1322 - 1329	472.7277	943.4408	943.4399	1	0	R.DSYGYLAR.A	(Ions score 31)
1322 - 1329	472.7277	943.4408	943.4399	1	0	R.DSYGYLAR.A	(Ions score 33)
1344 - 1359	867.4592	1732.9039	1732.9182	-8	0	R.VLNPFEALTTMVTAAR.T	(Ions score 2)
1344 - 1359	578.6468	1732.9184	1732.9182	0	0	R.VLNPFEALTTMVTAAR.T	(Ions score 30)
1367 - 1379	655.9098	1309.8050	1309.7969	6	0	R.SNLVPLLLGSAVK.R	(Ions score 14)
1367 - 1379	655.9098	1309.8050	1309.7969	6	0	R.SNLVPLLLGSAVK.R	(Ions score 45)
1367 - 1379	655.9098	1309.8050	1309.7969	6	0	R.SNLVPLLLGSAVK.R	(Ions score 37)
1416 - 1424	479.2872	956.5598	956.5542	6	0	R.AVTVEPTIK.S	(Ions score 11)
1479 - 1486	436.7465	871.4785	871.4763	3	0	R.DADLLGLR.F	(Ions score 18)
1507 - 1516	515.3055	1028.5964	1028.5978	-1	1	K.TRAPSGVLTK.Y	(Ions score 9)
1509 - 1516	386.7306	771.4467	771.4491	-3	0	R.APSGVLTK.Y	(Ions score 4)
1509 - 1516	386.7306	771.4467	771.4491	-3	0	R.APSGVLTK.Y	(Ions score 13)
1517 - 1524	458.7982	915.5818	915.5793	3	0	K.YPLLVLAK.G	(Ions score 15)
1517 - 1524	458.7982	915.5818	915.5793	3	0	K.YPLLVLAK.G	(Ions score 17)
1517 - 1524	458.7982	915.5818	915.5793	3	0	K.YPLLVLAK.G	(Ions score 16)
1525 - 1533	506.7996	1011.5847	1011.5825	2	1	K.GRLPETALR.V	(Ions score 16)

Figure S3

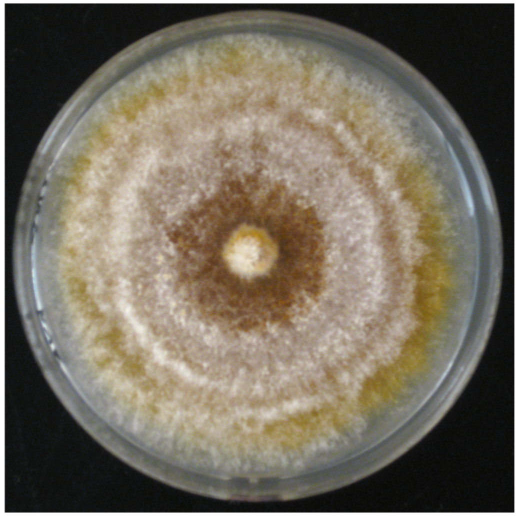

EP155/VF

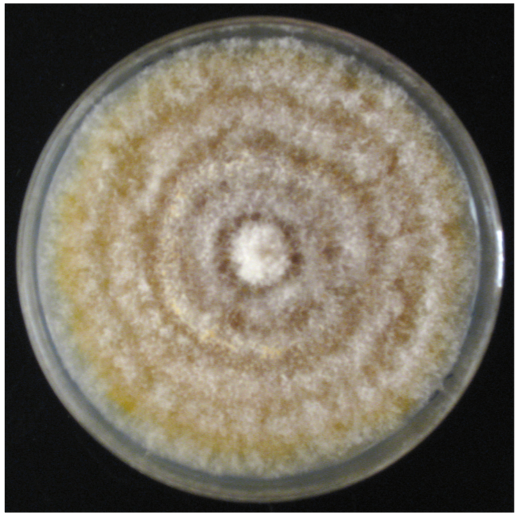

EP155/AalVV1

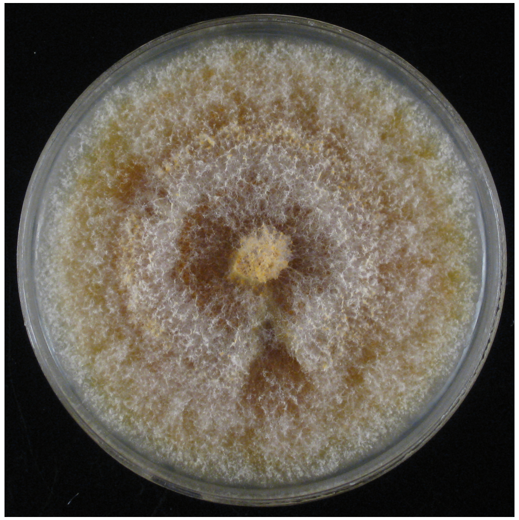

Δ*dcl2*/VF

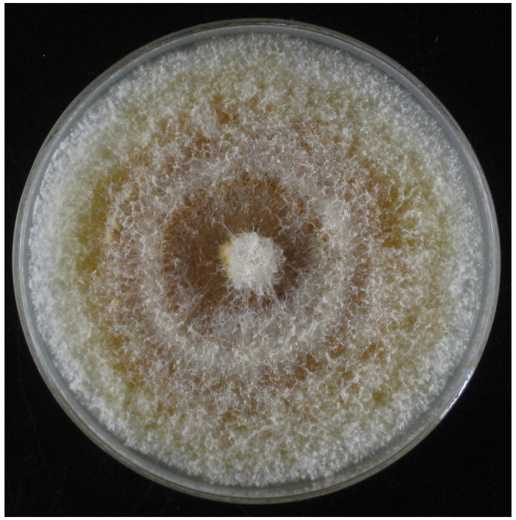

Δ*dcl2*/AalVV1

Figure S4

Phomopsis vexans RNA virus KP090346

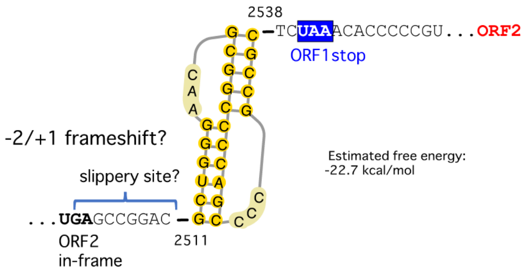

Nigrospora oryzae victorivirus 1 KT428155

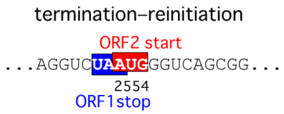

Supplement: Supplementary file 1 [file viruses-11-00577-s001.pdf]
